# Notes and News

**Published:** 1887-09-03

**Authors:** 


### NOTES AND NEWS.

COLLECTED AND COMMUNICATED.

A NEW workhouse infirmary has been built at Worcester from the designs of Messrs. Rowe and Son.

THE North Cambridge Hospital at Wisbech is now free, and the Friendly Societies of that town are proposing to initiate a movement for the purpose of renewing their contributions to the funds.

THE Farsley Local Board have decided that one infectious hospital of sufficient accommodation, with an efficient staff, will best meet the requirements of the North Bierley Union.

IT is proposed to spend £2,000 in the enlargement of the Free Providence Hospital at St. Helen's. Lord Stanley has consented to perform the inaugural ceremony.

THE street collection taken on Saturday and Sunday last on behalf of the Bournemouth Hospital, under the auspices of the United Friendly Societies, amounted to a little over £50.

THERE is a deficiency of hospital accommodation at Sheffield for infectious cases. The borough hospital committee held a meeting on the 23rd ult., Alderman Brooksbank presiding, for the purpose of further considering the question of providing accommodation for small-pox patients. So far as is known no definite conclusions were arrived at.

THE town of Hull collected last year £691 16s. 8d. in its churches and chapels in aid of the local hospital. In 1885, £738 15s. 8d. was the amount received. There was thus a falling off in 1886 of nearly £50. The larger sum is unworthy of the wealth and population of Hull; the smaller amount is disgraceful.

THE people of Huntley have collected about £2,000 in aid of their proposed cottage hospital. They are now arguing and disputing about sites, life members, etc., etc. It is to be hoped that as the result of all this talk the hospital may be a visible object on the landscape within a reasonable period.

THE present epidemic of fever in the metropolis is assuming larger proportions. Last week 820 patients were lying in the different hospitals of the Metropolitan Asylums Board. Of these 745 were suffering from scarlet fever, one from typhus, 68 from enteric, and six from other diseases. The majority of the patients seem to be drawn from the middle and lower classes. The number of scarlet fever cases continues to increase.

THE various places of worship at Lowestoft have set a noble example to other churches and chapels in the amounts contributed on their local Hospital Sunday. The sum collected at St. John's Church alone amounted to £92 12s. If the London congregations were to follow this liberal example the £100,000 so often asked for would speedily be reached.

THE *British Medical Journal* expresses the opinion that a provident system for nurses upon a sound actuarial basis would be of the greatest possible advantage to them, and goes on to say that though such an institution, if once the advantages it might offer could become widely known, might quickly become to a large extent self-supporting, yet there would always be scope for external assistance, and probably no more useful destination could be found for a part, at least, of that Jubilee Fund which may be known in future as Queen Victoria's Bounty.

A CORRESPONDENT of the *Buxton Herald* says that Mr. John Mason, who took a noble part in aid of the distressed Lancashire operatives during the cotton famine, and who has since done much in obtaining similar aid for the unemployed workmen in the east of London, is now lying ill in the Devonshire Hospital, Buxton. Mr. Mason has lately suffered from a severe reverse of fortune, and is stated to be in very poor circumstances.

ON Saturday last the Treasurer and Secretary of the Total Abstinence Sons of the Phœnix paid into the funds of the Royal Hospital for Diseases of the Chest the sum of £28, being the amount collected at a church-parade on the previous Sunday, at the Church of St. Philip, Clerkenwell. This amount, added to the results of previous church-parades held by the Sons of the Phœnix during the past two years, makes a total of £302 collected by them and handed to this hospital in aid of its greatly increased annual expenditure, caused by the erection of the new wing, containing eighty beds.

THE following vacancies are announced this week, the amount of the salary being placed in each case after the name of the institution :—

**Resident Medical Officers.**

Brighton and Hove Lying-in Institution.—£120.
Bristol Royal Infirmary. Assistant Medical Officer.—£80.
General Hospital, Birmingham. House Governor.—£300.
Hulme Dispensary. House Surgeon.—£130.
Memorial Hospital, Jarrow. House Surgeon.—£130, £150, £175.
Royal Berks Hospital.—No Salary.

**Non-resident Medical Officers.**

Bristol Royal Infirmary. Obstetric Physician.
Gainsborough Friendly Societies.—£160.
Provincial School. Demonstrator of Anatomy.
Weston-Super-Mare Provident Dispensary.—£60.

**Trained Nurses.**

Bolton Infirmary. Charge for Male Wards, £25 to £30.
Bolton Infirmary. Assistant Nurse.
Central London Throat and Ear Hospital.—£20.
Eastern Fever Hospital, The Grove, Homerton, E.—£30 to £36.
Kingston Home, Baker Street, Hull. Several.
North-Western Fever Hospital. Several.—£30 to £36.
Trained Nurse for Small Hospital. Apply, Matron, Central London Throat and Ear Hospital.
Weston-Super-Mare Hospital. Night Nurse.—£25.

---

DR. GEORGE A. ATKINSON has been appointed honorary physician to the Newcastle Dispensary. The other candidates were Dr. Baumgartner and Dr. Hugo Hardcastle.

---

NO sooner has the Tavistock Cottage Hospital been established and opened, than it has come into immediate requisition. A fire broke out in the oil-room at the Great Western Station in the town, doing great damage. Two men, Warren and Waldron, were seriously injured during the conflagration, and are both in the hospital. The institution owes its completion at any rate, if not its inception, to the Jubilee year. The subject of a Cottage Hospital has been for some time under consideration, and the Duke of Bedford headed the subscription list with a handsome donation of £300. Mr. D. Radford gave £200, and other inhabitants of the district were equally generous. The hospital was opened by Mr. Radford free from debt, and with an endowment fund of £1,000.

---

A "JOHN WILLIAMSON" ward has been christened in commemoration of the late Alderman John Williamson, J.P., at the South Shields Infirmary. Mr. Williamson laid the foundation stone of the present buildings in 1871, and from that time until his death was president of the institution. During the last fifteen years he has contributed the munificent sum of over £3,000 to its funds.

---

HONORARY hospital appointments are almost always eagerly sought after, for reasons which are very well known. The methods of appointment used are by no means always the best. A typical case is now occurring in connection with the Dorset County Hospital. A vacancy has recently been declared; there are about 960 voters entitled to take part in the appointment; few of these know anything whatever about the merits of the respective candidates, and yet every one of them must be canvassed, and thoroughly canvassed, by any candidate who wishes to be successful. This is by no means as it should be, and sooner or later radical changes will have to be made.

---

ALDERMAN RICHARDSON, J.P., Mayor of Stockton, presided at the annual general meeting of the governors of the hospital on the 23rd ult. The report, which was read by Mr. John Settle, the secretary, expressed deep regret at the death of the late mayoress. During the year important sanitary improvements had been effected in the hospital. The financial statement showed that the receipts had been nearly £100 in excess of those of the previous year.

---

DR. RUSSELL, who, it appears, is now acting as assistant to Dr. Dewar, has been appointed one of the medical officers to the Arbroath Infirmary. "Justice," writing to the *Dundee Advertiser*, asks, "Should he, an assistant to Dr. Dewar, be appointed to such an office, to the exclusion of other medical men in practice for themselves and resident in the town, and also applicants for the appointment? I don't think so, and people generally don't think so."

---

THE Gloucester Free Hospital for the children of the poor is in great straits for want of funds. About 900 out-patients, chiefly from Gloucester and the neighbourhood, and 200 in-patients from the city and all parts of the country, are treated annually. It is said that if the necessary funds are not immediately forthcoming one wing of the institution will have to be closed. Unless there is some explanation to be offered this state of matters must be considered highly discreditable to the rich county of Gloucester.

---

THE Mayor of Birmingham, Sir Thomas Martineau, presided at a meeting on behalf of the scheme for the improvement of the Queen's Hospital. There was a large and influential attendance. It is proposed to extend the present buildings, and to raise the older portion to a higher sanitary level. The sum of £10,000 is mentioned as the probable cost of the suggested improvements, and an appeal for subscriptions will be issued forthwith. The Birmingham managers are well advised in undertaking a much-needed work, and there should be no difficulty in raising the necessary funds within a reasonable time.

---

THE Rev. Sir George Wilmot-Horton, Bart., presided on Monday at the annual meeting of the subscribers to the Barton-under-Needwood Cottage Hospital. Lady Horton, Mr. and Mrs. Palmer, Mr. and Mrs. Knight, Mr. and Mrs. Ludlam, Mr. and Mrs. Reeve, and others, were also present. For the first time in the eight years during which the hospital has been at work, the income has been less than the expenditure. This, too, in the Jubilee year! Since 1880 there has been a steady diminution in annual subscriptions. At that period these amounted to £165 7s., now they are no more than £127 19s. 6d., a falling off of nearly twenty-five per cent. Perhaps the numbers of the more prosperous inhabitants have diminished during that period. It would be a curious, possibly an impertinent question, whether or not the cost of the ladies' wardrobes at Barton-under-Needwood has exhibited a corresponding diminution in the same period? Will anybody who knows whisper in our ears the facts as to this latter point?

It is said that a portion of the ground hitherto used as a recreation ground by the convalescent patients at the Station Hospital, Southsea, has been lately converted into a "tennis level," and labelled "sacred." A correspondent of the *Hampshire Telegraph* says he has "seen from twelve to sixteen youngsters arrive on the ground fully equipped for tennis, but to whom a game of 'tig' or a romp round the ground seemed to be infinitely more attractive." He professes his ignorance as to who is the authority who may be responsible for this change, but considers it a great hardship that the patients should be shut out from a piece of ground which was originally intended for their exclusive use.

---

"Barnsley Feast" is a venerable institution, and holds its ground manfully. The hospital committee of the town have this year taken advantage of the general holiday-making to hold their annual festival. At Barnsley the Hospital Saturday and Sunday collections provide nearly half the annual income of the institution. The proceedings opened with a drum-head service at the Queen's Grounds. The Volunteers and Yeomanry assembled on Market Hill, from whence they marched to church. The Rev. W. W. Kirby, M.A., rural dean and chaplain to the Force, preached the sermon. In the afternoon a large open-air musical festival was held. The Mayor, Alderman Marsden, presided, and the Rev. J. L. Brereton, M.A., Vicar of St. Peter's, opened with prayer. There were about 400 performers, Mr. A. Gill, of Barnsley, conducting. Special trains were run from Wakefield and other surrounding towns and villages, and the town was crowded for many hours. Collections were taken at the churches and chapels, and at the open-air concert, on behalf of the hospital funds.

---

Lancaster bears a historical name, and is a town of no mean antiquity. Ancient fame and lineage demand honourable character and conduct, as well in towns as in men. *Noblesse oblige* is a principle of universal application. The Rural Sanitary Authority is displaying tactics to which the very unpleasant term of "shuffling" may not very inappropriately be applied. Nature and circumstances have made Lancaster the centre and head of an important district, and the Lancastrians are doing their utmost to avoid the responsibilities which always devolve upon the head. A hospital for infectious diseases is needed for the district. The Sanitary Authorities have the power to erect one : and they are the proper persons to do it. If they are not—who are? In order to evade their responsibilities they are taking advantage of the infirmary scheme which is in course of incubation, and are proposing to tack on the infectious hospital to the new infirmary, if the latter should ever be built. They ought to know better. Although an infectious hospital is no great source of danger to a locality, it ought never to constitute a portion of a general hospital, except in cases of the most unquestioned necessity. Nobody can plead that such is the case at Lancaster. The Rural Authorities must cease from their peddling, and approach the subject like sensible and far-seeing men. The spirit of the vestry is out of date, and must be exorcised and relegated to the museum of local fossils with all possible promptitude.

The provinces are far ahead of London in the versatility of the efforts they put forth on behalf of their hospitals. A festival at Holbeck draws together twenty thousand people, and a Flower Show at Redditch delights young and old. The annual Bredon Flower Show on behalf of the Birmingham hospitals was held on Saturday, and was in every respect a decided success. Five years ago the best room at the "Wagon and Horses" Inn was sufficient to accommodate both flowers and visitors. Now two large marquees are all too small. Such is the popularity of hospitals combined with flower-shows. Londoners combine their hospitals with dull speeches and spiritless indoor entertainments. Nobody can digest a hospital sandwiched between such bread and butter as that. Voluntary hospitals are the pride and delight of Britons everywhere, but if they are to survive and grow with the people they must be popularised in every legitimate way. Hospital managers must take off the grandmothers' nightcaps they have worn so long, and which are quite out of date ; and they must don the bright head-millinery of the period, if they want to be looked at. A word to the wise is enough. Are hospital managers wise?

---

Mr. William White, referring to a note on small-pox in The Hospital for August 20, writes :—"You wish to know how anti-vaccinators explain the absence of small-pox from London. Why should they try to explain it? They might point out that if small-pox is absent, scarlet fever is present, and may be its substitute. They might also inquire how it was that in 1871-72, with London as well vaccinated as at the present day, there occurred the severest small-pox epidemic of the century, when 9,657 perished. The appearances and disappearances of certain maladies are mysterious, but this seems certain that the average prevalence of zymotic affections is determined by the insanitary conditions of the community in which they are exhibited ; and that it is probable that these affections are interchangeable. Thus Dr. Watt discovered in 1813 that when small-pox abated in Glasgow, the tale of mortality was kept up chiefly by measles and whooping-cough. Moreover, it is the habit of small-pox to disappear and reappear. Thus Des Gouttes, secretary to the Syndic of Geneva, writing to Dr. Haygarth in 1791, observed : 'An epidemic of small-pox is of almost regular occurrence every five years ; and between the epidemics it frequently happens that we have no small-pox whatever, either in Geneva or its vicinity.' Dr. Waterhouse, 'the Jenner of the New World,' writing in 1787 from Boston, where small-pox was often prevalent, said : 'I do not believe there is at present a single person infected by small-pox in all the four New England Governments, that is, not one in a million of people.' In Philadelphia not a death from small-pox occurred in the years 1812, 1813, 1814, 1815, 1820, 1821, 1822, and 1878 ; whereas there died of that disease 325 in 1824, 427 in 1852, 758 in 1861, 524 in 1865, 1,879 in 1871, 2,585 in 1872, and 1,336 in 1881. Who can explain these erratic manifestations? Small-pox, like typhus, is remarkably amenable to sanitation. Indeed, I question if an epidemic ever occurred in a community where the conditions of health were fairly good. The sanitary improvements effected in London, and going forward, ought to make of small-pox a less and less common disease."

